# Perceived Barriers to Application of Glycaemic Index: Valid Concerns or Lost in Translation?

**DOI:** 10.3390/nu3030330

**Published:** 2011-02-28

**Authors:** Shannan M. Grant, Thomas M. S. Wolever

**Affiliations:** 1 Department of Nutritional Sciences, University of Toronto, 150 College Street, Toronto, Ontario, M5S 3E2, Canada; 2 Division of Endocrinology and Metabolism, Department of Medicine, St. Michael’s Hospital, 61 Queen Street East, Toronto, Ontario, M5C 2T2, Canada; Email: thomas.wolever@utoronto.ca

**Keywords:** glycaemic-index, knowledge-translation, education, clinician, dietitian, barrier(s)

## Abstract

The term glycaemic-index (GI) originally appeared in the literature in the early 1980s. GI categorizes carbohydrate according to glycaemic effect postprandially. Since its inception, GI has obtained and maintained interest of academics and clinicians globally. Upon review of GI literature, it becomes clear that the clinical utility of GI is a source of controversy. Can and should GI be applied clinically? There are academics and clinicians on both sides of the argument. Certainly, this controversy has been a stimulus for the evolution of GI methodology and application research, but may also negatively impact clinicians’ perception of GI if misunderstood. This article reviews two assessments of GI that are often listed as barriers to application; the GI concept is (1) too complex and (2) too difficult for clients to apply. The literature reviewed does not support the majority of purported barriers, but does indicate that there is a call from clinicians for more and improved GI education tools and clinician GI education. The literature indicates that the Registered Dietitian (RD) can play a key role in GI knowledge translation; from research to application. Research is warranted to assess GI education tool and knowledge needs of clinicians and the clients they serve.

## 1. Introduction

Glycaemic-index (GI) first appeared in the literature in the early 1980s as a means by which to categorize carbohydrate according to glycaemic effect postprandially [1,2]. Carbohydrate containing foods can be categorized according to the following GI classes: low-GI (<55); medium GI (55 to 69) and high GI (≥70) [3]. The GI is based upon a glucose (reference) scale where glucose has a GI of 100 (using standardized methodology). For instance, the high GI cut point can be expressed as 70/100 [4]. Postprandially, starchy foods included in the low-GI category are absorbed more slowly across the intestine than medium or high GI foods. Slow postprandial intestinal absorption of starchy low-GI food results in a gradual increase in blood glucose (BG) and lower peak BG when compared to the prominent peak in BG observed after consuming a high GI food [2,4,5]. A large body of data suggests a diet composed of low-GI food has a role to play in the prevention or treatment of a number of chronic conditions including type 2 diabetes mellitus (T2DM), cardiovascular disease, and cancer [6,7,8,9,10,11,12]. Despite the existence of these supporting data, the utility of GI continues to be a topic of debate. 

Clinicians, more specifically Registered Dietitians (RD), continue to question the utility of the GI concept in their practice [13]. A postal cross-sectional survey was conducted in Canada (2003), including members of the Dietitians of Canada (DC) and Ordre Professionnel des Dietetistes du Quebec (OPDQ). This questionnaire was created to assess how many RDs identify as “users” or “non-users” of the GI concept, what RDs perceive to be benefits of and barriers to GI utility, RDs general knowledge of GI, and their confidence in teaching the concept [13]. A total of 6060 RDs (DC = 4014; OPDQ = 2046) were originally contacted. Of the total respondents (*n* = 2857), 40% (*n* = 724) identified as users, while 60% (*n* = 1,081) identified as non-users. One thousand and fifty-seven respondents reported treating patients with diabetes mellitus (DM). Of this subset, 39% (*n* = 415) identified as users and 61% (*n* = 642) as non-users. Only 3% of respondents (*n* = 642) were unaware of the GI concept. Of members who identified as users, 90% used GI as a general descriptor of carbohydrate absorption (fast [high GI] *versus* slow [low-GI]) and 56% reported teaching GI with the aim of facilitating glycaemic control. The following three barriers were most commonly selected by non-users working with clients living with DM: (1) 57% felt the GI concept was too complex for patients to understand, (2) 46% reported lack of education resources, (3) 31% did not know how to use the concept. Of the study participants, those who identified as users were more likely to have a greater DM caseload and perceive benefits to and confidence in teaching the GI concept. 

## 2. GI Knowledge Translation

Knowledge translation is a term used by health researchers to describe the relationship between knowledge creation and application [14]. The translation of knowledge discovery into practice applications or policy is a fundamental component of the knowledge-to-action process. In the current research environment, agencies supporting health research do not only expect research be published in peer-reviewed journals, but also that plans will be outlined, within the original grant proposal, to translate the research findings into practice or policy [14]. A rather new concept among researchers, this concept may then inspire the question, “who should take responsibility for the end use of findings?” [15]. 

As outlined in the literature, clinicians, specifically RDs, can play a key role in facilitating GI education [13,15,16,17,18]. It is therefore important that RDs perceived barriers to GI utility be addressed by researchers. Studies have shown that RDs that work with people living with DM ≥ 11 years are more likely to use the GI concept [13,19]. Research has also shown that outpatient RDs are more likely than in-patient clinicians to use GI education [13]. Research looking at why these differences exist, whether or not perceived barriers to GI utility are valid, and how to overcome these barriers are key to facilitating GI knowledge translation and continuing the knowledge-to-action cycle. 

## 3. Perceived Barriers to Knowledge Translation

The perceived barriers to GI utility highlighted in Kalergis *et al.* (2006) are recurrently noted by GI critics within and outside of Canada [13,19,20,21,22]. Inspired by these works, this article will review the three aforementioned barriers along with other commonly documented barriers to GI application. Many of these barriers have been used as justification for the assessment that GI is too difficult for clinicians and/or nutrition professionals to teach and is too difficult for clients to understand and apply. The reviewed literature will, therefore, be organized under the following headings/themes: (1) The GI concept is too complex (for RDs to teach and clients to understand) and (2) The GI concept is too difficult for clients to apply. The following paragraphs will show that perceived barriers to GI utility are, in many cases, unfounded or easily addressed. Despite this, these criticisms are ever-present in peer-reviewed literature and popular media; literature clinicians utilize to make professional judgements.

### 3.1. Assessment 1: The GI Concept Is Too Complex (for RDs to Teach and Clients to Understand)

The perceived complexity of GI has been repeatedly noted as a barrier to GI utility. The assessment that the GI concept is too complex is often supported by the following three barriers or criticisms: GI education opposes current dietary guidelines (and is therefore confusing), GI terminology is confusing and there is a shortage of GI education materials [13,19,20,21,22]. This section will examine these criticisms using available literature. 

The Canadian Diabetes Association (CDA) (2008) (Clinical Practice Guidelines) recommends that GI education be used as a supplement to standard care with use varying by client interest, ability and need. GI education, therefore, should be presented as a supplement to dietary recommendations; not as an alternative to it [23]. Current North American dietary recommendations include suggestions regarding serving/portion size (varying by sex and stage of life), fibre, fat and sugar intake and promotes dietary variation/diversity and moderation. GI education focuses primarily on the concept of carbohydrate quality and glycaemic response [1,2,24]. Despite the existence and availability of this information, GI critics seem confident that the low-GI diet encourages increased use of foods high in fat and sugar and promotes an increase in energy consumption [20,22]. The existence of this misinformation is not surprising, especially in light of the recent finding that only 61% of Canadian RDs who identify as GI education users and 26% of non-users report being aware of CDAs position on GI [13]. A number of studies display that low-GI foods can be consumed as part of a diet based on current dietary recommendations. For instance, Grant *et al.* [25] used low-GI education as a supplement to standard care to obtain improved glycaemic control in women with gestational diabetes mellitus and impaired glucose tolerance. Addition of low-GI education did not result in divergence from current dietary recommendations in this group; energy and macronutrient intake was matched to controls post intervention. Moreover, Frost *et al.* [26] facilitated adherence to current dietary recommendations using low-GI education. In this study, participants on the low-GI diet consumed less dietary fat and more fibre. Similar results have been found by others [27,28,29,30].

To encourage efficient GI knowledge translation, clinicians and academics must work together to efficiently translate scientific jargon and/or concepts. This involves establishing appropriate translations and audience-appropriate phraseology; whether the audience is composed of researchers and clinicians, individual clients or whole populations [31,32,33,34,35]. Slabber [36] offered examples of such translations accompanied by client-focused education tools and phraseology, concluding that GI terminology need not be any more difficult than teaching other concepts included in standard medical nutrition therapy. For instance, low and high GI can be explained using terms like, “slow and fast acting carbohydrate”. “Retrogradation” can be explained using the following phrasing: “When cooked (red) potatoes are cooled in the fridge, the starch in them becomes sticky and gel-like.” Notwithstanding, Mendes *et al.* [19] found that GI was not deemed appropriate for clinical use by 78% of American RDs treating children for obesity. RDs in this study indicated they felt knowledgeable about GI (77%; *n* = 92), but felt GI terminology/concepts were too challenging for study participants. Although these data were published after the publication of the following statement of the American Diabetes Association (ADA) (2005): “…use of GI can provide additional benefit over that observed when total carbohydrate is considered alone…”, American health agencies traditionally were in opposition to GI utility [21,37,38]. This traditional position most likely affected the sample’s (American RDs’) perception of GI application. Conversely, Frost *et al.* [26] showed that people can successfully and significantly lower diet-GI after verbal and written communication. Similar success has been noted by others [28,39]. 

Canadian RDs believe a shortage of GI education materials is a barrier to GI utility [13]. Clinicians and/or researchers have set out to address this resource-gap in an attempt to overcome this barrier to utility. For instance, the Canadian Diabetes Association (CDA) has published a Glycaemic-Index Education Tool that can be downloaded off the CDA website [3]. The CDA education tool includes substitution lists for low, medium and high GI food choices frequently used in the Canadian diet. The following GI categories are included in this tool: Low-GI (55 or less; choose most often), medium GI (56–69; choose more often) and high GI (70 or more; choose less often). The CDA tool format is based upon recommendations from clinical scientists, basic scientists and diabetes educators of how to best provide GI information to clients. This tool also summarizes current dietary recommendations and key take-home messages for DM management; demonstrating GI as a supplement to standard care. Variations of the low-GI food substitution list are available in peer‑reviewed literature, popular media, and online [40,41,42,43,44]. Food substitution lists have been used in our laboratory and others to achieve a moderate difference in food choice and to obtain a significant decrease of dietary GI [45,46]. Although a very useful tool, the CDA GI education tool only lists 12–18 foods under each heading and does not include a section under each GI category for the RD to add individualized food substitutions. 

American and South African RDs report a lack of usable/suitable GI teaching tools as a barrier to GI utility; rather than a lack of tools [18,19]. *Understanding the Glycaemic Index* is a Canadian GI education tool that illustrates that similar findings may be obtained in Canada [47]. This tool, developed by clinicians and researchers, has been published on the Canadian Sugar Institute website and was mailed out to RDs across Canada. Although this tool contains some very useful information, there are conceptual errors on this tool and therefore misinformation. For instance, the following statement is included in this tool “Fat or protein eaten along with carbohydrate… reduces the GI of the carbohydrate”. This statement is incorrect and reflects back to the aforementioned GI myth that low-GI education encourages increased use of fat. The authors of this tool have mistakenly used the term glycaemic index when they are describing glycaemic response; highlighting and propagating a hiccup in the knowledge translation process. GI is a characteristic of available carbohydrate and is not synonymous with glycaemic response [2,4]. Protein and fat have effects on glycaemic response which are independent of those produced by carbohydrates and occur by different mechanisms [4,48,49]. The proper terminology would be, “Adding fat and protein to a carbohydrate reduced the glycaemic response”. This education tool provides an example of how confusion, secondary to GI terminology and phraseology, may exist among clinicians and/or researchers. Research assessing clinicians’ comprehension of GI concepts and terminology and examining GI misinformation in academic literature, websites and popular media are warranted.

### 3.2. Assessment 2: The GI Concept Is Too Difficult for Clients to Apply

There have been a number of barriers cited in the literature to support the assessment that the GI concept is difficult for clients to apply. Two examples of these proposed barriers include: The low-GI diet limits food choice and GI is not accepted by clients. Although noteworthy barriers to any dietary change these barriers are not currently supported by the existing data on GI. The following paragraphs will review these data.

It is unrealistic for a clinician to expect clients to consume low-GI carbohydrate 100% of the time [36,43,50]. The literature has identified this expectation as a limitation and an example of how low-GI limits food choice [20,21,38]. Conversely, the literature indicates that GI “users” recognize that medium to high GI foods may be appropriate in some cases and flexibility is important for sustainable lifestyle change [26,27,43,50,51,52,53]. Moreover, research shows one need only consume low-GI choices 50–60% of the time to obtain a significant reduction in dietary GI (7 to 11 units) and/or documented benefits [4,50]. Despite this, the following two barriers to low-GI diet compliance were noted by Brekke *et al.* [54]: lack of choice when dining out and lack of ideas when cooking at home. It is important to note, however, that this criticism is not unique to the low-GI diet. Similar barriers have been noted during dietary interventions that do not use GI education [52,53,54,55,56]. In general, it is challenging for one to change lifestyle behaviours from initiation to maintenance [52,53,57,58]. To address these concerns, clinical scientists (including RDs) in our laboratory and others have designed culturally sensitive GI recipe booklets, GI food lists and tips for dining out that outline low, medium and high GI foods [3,25,36,40,43]. 

The question of whether or not low-GI is accepted by clients has fuelled much research and is ever‑present in GI literature. Once a clinician makes the judgement call that the literature supporting a dietary intervention is adequate, he/she often then asks the questions, “will clients eat the recommended food?” and “is this lifestyle change sustainable?”. There is a wealth of literature that indicates the answer to both questions is yes in the context of a low-GI diet. For instance, Burani and Longo [59] assessed the effect of low-GI medical-nutrition-therapy (MNT) on multi-ethnic American adults living with type 1 and 2 DM (*n* = 21) one year after education. At baseline 90% of the participants reported not understanding GI and 19% reported feeling they could include it in their current lifestyle. Post-intervention, average dietary GI was 45 and a statistically significant decrease in GI was achieved by 95% participants. Moreover, post-intervention, 85% of participants reported they had adequate understanding of GI and 95% felt they possessed enough knowledge to apply it in their lifestyle after the study. All study participants accepted GI education as a useful supplement to current dietary recommendations, perceived low-GI foods to be healthy and reported beneficial effects on glycaemic control and weight management. Perhaps most important to note, 100% of participants reported planning to include low-GI as a permanent lifestyle change; indicative of low-GI diet acceptability and sustainability in this sample. These findings are in agreement with those of a recent prospective randomized control trial conducted in Australia where pregnant women following a low‑GI diet were more likely to agree their study diet was easier to follow in comparison to those on a medium-to-high GI diet [51,59]. 

Comparable results have also been seen in children and young adults [60,61]. For instance, using a cross-over design, Nansel *et al*. [60] looked at low-GI food acceptability of standard[S] *versus* low‑GI[LGI] menus in a youth camp for children with type 1 and 2 DM in the United States. Food service staff were provided with low-GI food and cooking instructions aimed to facilitate creation of a low-GI menu. Camp kitchen staff reported low-GI foods were acceptable in terms of preparation effort, perceived healthiness and youth appeal. A questionnaire (Likert Scale format; 1 = “I didn’t like it at all”, 5 = “I liked it a whole lot”) was provided to the children after every meal and evening snack. Camp attendees (*n* = 140; age 7 to 16) provided comparable ratings for low-GI food and standard foods served at dinner[D] and snacks[SNK] (D = 3.68 [LGI] *vs.* 3.79 [S], *p* = 0.30; SNK = 3.74 [LGI] *vs.* 3.79 [S], *p* = 0.60). On the other hand, low-GI foods at breakfast[BFST] and lunch[L] were acceptable, but were rated lower than standard foods (BFST = 3.76 [LGI] *vs.* 4.04 [S], *p* = 0.01; L = 3.64 [LGI] *vs.* 3.88 [S], *p* = 0.01). Similar findings were obtained during a previous long term prospective randomized trial in children living with T1DM. In this study, children on a low-GI diet did not decrease dietary quality or choice in comparison to a control group using traditional carbohydrate exchange dietary advice [36,39].

## 4. Glycaemic Index: Breaking down the Barriers

In Canada, the question of whether to use or not to use GI education as part of medical nutrition therapy for diabetes prevention and treatment is left to the discretion of the clinician. Clinicians often look to colleagues, key journal articles, education tools and online media for information when deciding which therapies to utilize. It is therefore important that reliable literature and education materials/tools are easily available to keep interested practitioners well-informed. Propagation of GI mythology represents a barrier to GI application and an interruption of the scientific process. An aim of this article is to address and move beyond commonly cited GI mythology, while inspiring research and development that will test GI utility and highlight and overcome valid barriers to GI application. Clinicians, specifically Registered Dietitians (RDs), can play a key role in facilitating GI knowledge translation from laboratory to client. It is therefore important that RDs’ perceived barriers to GI utility are studied and RDs be included in the research process.

While GI research continues and data on GI-utility and/or methodology are collected, there is a need for ongoing review of perceived and actual barriers to GI utility from the perspective of the educator and the client. Many of the perceived barriers reviewed in this paper may not be currently supported by existing data, but indicate that supplementary research is warranted. Research on clinicians’ and clients’ perceptions, knowledge of and application of GI is still needed to ensure that clinicians have access to adequate evidence-based literature on which to base their professional opinions. Existing data also support that there is a perceived deficiency in “reliable” GI education and/or reference materials available to clinicians. Review of existing GI education tools and, in some cases, development of new GI education tools for clinicians with the input of clinicians and clients is therefore warranted.
